# Mass Production of Virus-Like Particles Using Chloroplast Genetic Engineering for Highly Immunogenic Oral Vaccine Against Fish Disease

**DOI:** 10.3389/fpls.2021.717952

**Published:** 2021-08-23

**Authors:** Yoichi Nakahira, Kaori Mizuno, Hirofumi Yamashita, Minami Tsuchikura, Kaoru Takeuchi, Takashi Shiina, Hidemasa Kawakami

**Affiliations:** ^1^College of Agriculture, Ibaraki University, Ami, Japan; ^2^United Graduate School of Agricultural Science, Tokyo University of Agriculture and Technology, Fuchu, Japan; ^3^Ehime Fisheries Research Center, Ehime, Japan; ^4^Laboratory of Environmental Microbiology, Division of Basic Medicine, Faculty of Medicine, University of Tsukuba, Tsukuba, Japan; ^5^Graduate School of Life and Environmental Sciences, Kyoto Prefectural University, Kyoto, Japan; ^6^Department of Applied Biological Sciences, Faculty of Agriculture, Setsunan University, Hirakata, Japan

**Keywords:** red-spotted grouper nervous necrosis virus (RGNNV), virus-like particles (VLPs), chloroplast genetic engineering, oral vaccine, aquaculture

## Abstract

Nervous necrosis virus (NNV) is the causative agent of viral nervous necrosis (VNN), which is one of the most serious fish diseases leading to mass mortality in a wide range of fish species worldwide. Although a few injectable inactivated vaccines are commercially available, there is a need for more labor-saving, cost-effective, and fish-friendly immunization methods. The use of transgenic plants expressing pathogen-derived recombinant antigens as edible vaccines is an ideal way to meet these requirements. In this study, chloroplast genetic engineering was successfully utilized to overexpress the red-spotted grouper NNV capsid protein (RGNNV-CP). The RGNNV-CP accumulated at high levels in all young, mature, and old senescent leaves of transplastomic tobacco plants (averaging approximately 3 mg/g leaf fresh weight). The RGNNV-CP efficiently self-assembled into virus-like particles (RGNNV-VLPs) in the chloroplast stroma of the transgenic lines, which could be readily observed by *in situ* transmission electron microscopy. Furthermore, intraperitoneal injection and oral administration of the crudely purified protein extract containing chloroplast-derived RGNNV-VLPs provided the sevenband grouper fish with sufficient protection against RGNNV challenge, and its immunogenicity was comparable to that of a commercial injectable vaccine. These findings indicate that chloroplast-derived VLP vaccines may play a promising role in the prevention of various diseases, not only in fish but also in other animals, including humans.

## Introduction

According to a report by the Food and Agriculture Organization of the United Nations (FAO), global fish production reached 179 million tons in 2018, with first-hand sales estimated at USD 401 billion, and is projected to increase further over the next decade. In 2018, aquaculture accounted for 46% of global fish production (82 million tons), but its contribution is expected to be even greater in the future, as 34.2% of fish stocks are currently captured at biologically unsustainable levels (FAO, 2020). There are several factors that hinder the sustainable development of the aquaculture sector, but one of the major challenges is the prevention and control of infectious diseases. Disease outbreaks can cause enormous economic losses in the aquaculture industry. There are a wide range of pathogens that can cause serious diseases in aquaculture, including viruses, bacteria, protozoa, and metazoans (Lafferty et al., 2015). Antibiotics have been widely used in aquaculture to prevent and treat bacterial infections, but the overuse and misuse of antibiotics have led to the spread of antibiotic-resistant strains that pose health risks to humans (Reverter et al., 2020). In addition, antibiotics are ineffective against pathogens other than bacteria. Therefore, vaccines are better alternatives to antibiotics.

Viral nervous necrosis (VNN), also known as viral encephalopathy and retinopathy (VER) is one of the most serious infectious diseases in aquaculture. It affects a wide range of hosts, including 177 susceptible marine species, 62 of which have been reported to be prevalent (Bandín and Souto, 2020). VNN frequently causes mass mortality in the larval and juvenile stages of fish (Munday and Nakai, 1997), leading to serious economic losses. Therefore, the development of a highly immunogenic, cost-effective, and labor-saving VNN vaccine is of great interest to the aquaculture industry. The causative agent of VNN is the nervous necrosis virus (NNV), which belongs to the genus *Betanodavirus*. NNV is a small, non-enveloped icosahedral virus that contains two positive-sense single-stranded RNA molecules, RNA1 (3.1 kb) and RNA2 (1.4 kb), encoding the RNA-dependent RNA polymerase and the capsid protein respectively (Low et al., 2017). Based on the phylogenetic analysis of the partially variable sequences within the RNA2, betanodaviruses have been classified into four major genotypes: striped jack NNV (SJNNV), tiger puffer NNV (TPNNV), red-spotted grouper NNV (RGNNV), and barfin flounder NNV (BFNNV) (Nishizawa et al., 1997). Of these, RGNNV is the most prevalent and has the highest number of susceptible species (Bandín and Souto, 2020).

To date, several potential vaccines against VNN have been proposed, including inactivated vaccines, DNA vaccines, synthetic peptides derived from NNV capsid protein (NNV-CP), recombinant subunit vaccines, and virus-like particles (VLPs) (Costa and Thompson, 2016; Bandín and Souto, 2020). However, among them, only a few formalin-inactivated vaccines against the RGNNV genotype have been licensed, including one for sevenband grouper in Japan (Costa and Thompson, 2016) and two for sea bass in the Mediterranean market (Bandín and Souto, 2020). Commercially available injectable vaccines provide sufficient protective immunogenicity against VNN, but the parenteral method of vaccination is laborious, expensive, stresses the fish, and is not applicable to small fish in the larval or juvenile stages. In contrast, oral administration of vaccines is an ideal immunization method for fish because it is labor-saving, inexpensive, and fish-friendly, and can be applied to any size of fish. Although injection is the only recommended route of administration for all commercially available VNN vaccines, oral administration of a formalin-inactivated vaccine with a capsaicin adjuvant has been reported to confer significant protective immunity against VNN in fish (Gaafar et al., 2018). However, the use of capsaicin in combination with an inactivated VNN vaccine may not be feasible because currently available commercial vaccines are more expensive compared to the wholesale price of fish. Therefore, there is an impending need for alternative cost-effective vaccines.

VLPs are composed of one or more structural proteins that self-assemble to mimic the shape and size of the native virions but do not contain the viral genetic materials required for replication. VLPs are one of the most promising vaccine candidates because they are safer than infectious attenuated and inactivated vaccines and can effectively elicit humoral and cellular immune responses (Lua et al., 2014; Charlton Hume et al., 2019; Nooraei et al., 2021). To date, several VLP-based vaccines against human diseases are commercially available, including vaccines against hepatitis B virus (HBV), human papillomavirus (HPV), and hepatitis E virus (HEV) (Lua et al., 2014; Nooraei et al., 2021). In addition, the use of VLPs against animal diseases, including fish, is also being explored as a highly immunogenic and cost-effective vaccine candidate (Liu et al., 2012; Jeong et al., 2020).

NNV-CP is the only structural protein of NNV that can self-assemble into VLPs. Several potential VLP vaccines against VNN have been reported, including NNV-VLPs produced in insect cells using a baculovirus expression system (Lin et al., 2001; Thiéry et al., 2006), *Escherichia coli* (Liu et al., 2006; Lai et al., 2014; Lin et al., 2016; Chien et al., 2018), and yeast (Wi et al., 2015; Cho et al., 2017). Among them, orange-spotted grouper NNV (OSGNNV)-VLPs derived from *E. coli* (Chien et al., 2018) and RGNNV-VLPs expressed in yeast (Cho et al., 2017) have been demonstrated to confer protective immunity against NNV challenges in fish not only by injection but also by oral administration, indicating that recombinant VLPs could be promising oral vaccines against VNN. However, such a VLP vaccine is not commercially available yet. Since the microbial production of NNV-VLPs and the subsequent purification process are costly, an oral VLP vaccine may not be affordable.

Over the last three decades, plants have been utilized as bioreactors in the “plant molecular farming” process to produce biopharmaceuticals, such as antibodies, vaccines, growth factors, and cytokines (Lomonossoff and D’Aoust, 2016; Mahmood et al., 2020; Tusé et al., 2020). Although only a limited number of plant-derived recombinant biopharmaceuticals have been commercially approved, plant molecular farming offers several advantages over other bioreactors, including cost-effectiveness, scalability, and lower risk of contamination by bacterial endotoxins or animal pathogens (Lomonossoff and D’Aoust, 2016; Mahmood et al., 2020; Tusé et al., 2020). Plant-based protein expression systems can be divided into two types: conventional stable transformation, where transgenes are introduced into the nuclear or chloroplast genome, and transient gene expression, using *Agrobacterium*-mediated infiltration or plant virus-based vectors. Although generating stable transformants is time-consuming and labor-intensive, edible host plants expressing the desired recombinant antigen can be administered directly as oral vaccines without any purification process (Hernández et al., 2014). This is an attractive feature for the development of highly immunogenic, labor-saving, and cost-effective vaccines for animals, including fish (Clarke et al., 2013; Shahid and Daniell, 2016). Conversely, transient expression systems can produce recombinant proteins rapidly and in large quantities and are suitable to produce injectable vaccines that require the purification of recombinant antigens (Lomonossoff and D’Aoust, 2016; Mahmood et al., 2020; Tusé et al., 2020). Using transient expression systems, a variety of potential VLP-based vaccines against human diseases have been developed (Marsian and Lomonossoff, 2016; Rybicki, 2020), some of which are in clinical trials, including SARS-CoV2 vaccine candidates (Mahmood et al., 2020; Tusé et al., 2020; Shohag et al., 2021).

Taking advantage of plant molecular farming, it has been reported that transient expression of Atlantic cod NNV (ACNNV)-CP in *Nicotiana benthamiana* leaves causes the capsid protein to self-assemble into ACNNV-VLPs (Marsian et al., 2019). Furthermore, purified ACNNV-VLPs were able to partially protect sea bass from NNV challenge, suggesting that plant-derived NNV-VLPs could be candidates for VNN vaccines. However, the yield of ACNNV-VLPs obtained by transient expression in tobacco leaves was still low (10 mg of purified VLPs from 1 kg of fresh weight leaves), and the immunogenicity of plant-derived ACNNV-VLPs by oral administration has not yet been evaluated (Marsian et al., 2019).

Chloroplast genetic engineering (plastid transformation) has several advantages over nuclear transformation, the most distinctive of which is the ability to integrate transgenes into the entire highly polyploid chloroplast genomes (up to 10,000 copies per cell), resulting in extremely high levels of exogenous protein expression (Cardi et al., 2010; Jin and Daniell, 2015; Fuentes et al., 2018). In most cases, the accumulation levels of recombinant proteins expressed in transplastomic plants are 10–100 times higher than those expressed in nuclear transformants and can even reach more than 70% of the total soluble protein (TSP) (Oey et al., 2009; Castiglia et al., 2016). Further advantages of plastid transformation include the absence of positional effects and gene silencing, expression of multiple transgenes in an operon, and containment of the transgenes by maternal inheritance of the plastid genomes (Cardi et al., 2010; Jin and Daniell, 2015; Fuentes et al., 2018). Chloroplast genetic engineering has been applied to the production of high value-adding proteins, such as vaccine antigens, biopharmaceuticals, and industrial enzymes, as well as to the improvement of crop traits and metabolic engineering. In particular, various studies have reported the production of recombinant antigens derived from human and animal pathogens, and several studies have shown that oral administration of antigen vaccines expressed in crops with edible leaves, such as lettuce, can provide sufficient immunogenicity (Arlen et al., 2008; Davoodi-Semiromi et al., 2010; Yácono et al., 2012; Lakshmi et al., 2013; Daniell et al., 2019). Although examples of chloroplast-expressed VLPs are limited to those derived from human papillomavirus type 16 (HPV-16) (Fernández-San Millán et al., 2008; Lenzi et al., 2008; Waheed et al., 2011), dengue serotype 3 virus (Kanagaraj et al., 2011), and poliovirus serotype 2 (Daniell et al., 2019), it has been reported that HPV-16 L1 capsid protein accumulates at high levels (24% of TSP) in transplastomic tobacco plants and self-assembled into VLPs, which were highly immunogenic when injected intraperitoneally into mice (Fernández-San Millán et al., 2008). These results suggest that chloroplast genetic engineering may be a promising platform for the high-yield production of immunogenic VLP-based vaccines.

In this study, we generated transplastomic tobacco plants overexpressing the RGNNV capsid protein (RGNNV-CP), which could successfully self-assemble into VLPs (RGNNV-VLPs) in the chloroplast stroma. Both intraperitoneal injection and oral administration of chloroplast-derived RGNNV-VLPs to fish (sevenband grouper) showed high immunogenicity against NNV challenge.

## Materials and Methods

### Plant Growth Conditions

Tobacco (*Nicotiana tabacum* cv. Xanthi) plants were grown aseptically on Murashige-Skoog (MS) medium containing 3% (w/v) sucrose and 0.8% (w/v) agar at 25°C under a light–dark cycle with a 16-h light period (50 μmol photons m^−2^ s^−1^) and an 8-h dark period. Soil-grown tobacco plants were cultivated in a phytotron (Koitotoron SBH2515A, Koito Industries Co., Yokohama, Japan) using sunlight and natural daylengths with a temperature cycle of 12 h at 26°C and 12 h at 23°C.

### Construction of the Plastid Transformation Vector

To construct a universal plastid transformation vector for tobacco plants, the homologous DNA fragment corresponding to a part of the tobacco plastid *rrn16*-*rrn23* region [nucleotides 103441-107302 in *N. tabacum* chloroplast genome DNA (GenBank accession number Z00044.2)] was amplified by PCR using total cellular tobacco DNA as a template with the following primer pair: ADLF (5'-CACTCTGCTGGGCCGACACTGACAC-3') and ADLR (5'-CACTAGCCGACCTTGACCCCTGTT-3'), and inserted into *Pvu*II-digested pBluescript II KS (+) (Stratagene). The resulting plasmid was then digested with *Pvu*II, which recognizes the intergenic spacer region between tobacco plastid *trnI* and *trnA*, and the spectinomycin resistance gene (*aadA*), driven by the tobacco plastid P*rrn* promoter (nucleotides 102553-102728 in Z00044.2) and the bacteriophage T7 gene 10 leader sequence (G10L), was inserted into the *Pvu*II-digested site to obtain the universal plastid transformation vector, p16S-aadA-23S(T).

The cDNA encoding RGNNV-CP was derived from RNA2 of the RGNNV strain SG2001Nag (GenBank accession number AB373029). A DNA fragment consisting of the coding sequence of RGNNV-CP (nucleotides 27-1043 in AB373029) with codon usage optimized for plastid expression (*RGNNV-CPpt*) and the subsequent 3' non-coding region (3' NCR) from the RNA2 of RGNNV, flanked by *Nco*I and *Xba*I sites, was synthesized by Eurofins Genomics (Tokyo, Japan). The synthetic DNA fragment was cloned into the pPpsbA-TpsbA(R), harboring the tobacco plastid *psbA*-derived promoter and subsequent 5'-untranslated region (5'-UTR; complement of nucleotides 1595-1811 in Z00044.2; P*psbA*), a multiple cloning site, and 3'-UTR of tobacco *psbA* (complement of nucleotides 142-535 in Z00044.2; T*psbA*). The resulting expression cassette, P*psbA*::*RGNNV*-*CPpt*::RNA2-3' NCR::T*psbA*, was cut out with *Pst*I and *Sal*I and inserted into p16S-aadA-23S(T), which has cognate restriction sites just downstream of *aadA*, to obtain the plastid transformation vector, pRGNNV1. The nucleotide sequence of the final construct was confirmed by dye-terminator sequencing.

### Generation of Transplastomic Tobacco Plants

Plastid transformation was performed as described in previous studies (Nakahira et al., 2013; Kikuchi et al., 2018). Gold particles (0.6 μm) coated with pRGNNV1 plasmid DNA were delivered into young tobacco leaves cultured *in vitro* using the Biolistic^®^ PDS1000/He particle delivery system (Bio-Rad, Hercules, CA, United States). The bombarded leaves were kept in the dark at 25°C for 3 days, then cut into small pieces (~0.25 cm^2^), and placed onto RMOP agar medium [MS medium containing 3% (w/v) sucrose, 0.8% (w/v) agar, 0.1 mg/L 1-naphthaleneacetic acid, 1 mg/L N^6^-benzyladenine, 1 mg/L thiamine, 100 mg/L inositol] supplemented with 200 mg/L spectinomycin dihydrochloride as a selective agent. In tobacco plasmid transformation, it is common to use 500 mg/L spectinomycin dihydrochloride in all selection rounds, but we experienced low efficiency in acquiring resistant shoots or calli when 500 mg/L spectinomycin dihydrochloride was used in primary selection. Therefore, in our research group, the use of 200 mg/L spectinomycin dihydrochloride for primary selection was kept as a standard condition (Nakahira et al., 2013; Kikuchi et al., 2018). Resistant shoots and calli were transferred onto RMOP agar medium containing 500 mg/L spectinomycin dihydrochloride for the second selection. The obtained shoots were subjected to total cellular DNA extraction, as described previously (Kikuchi et al., 2018). To confirm that *RGNNV-CPpt* was correctly introduced into the specific region of the plastid genome, PCR-based genotyping was performed using the total cellular DNA as a template with the primer pair 1: ADL-F3 (5'-CGGGGGGGACCACCACGGCT-3') and ADL-R3 (5'-AGGGTTGAAGGGAGATAGTGCATCA-3'), and primer pair 2: RGNNV-Fd1 (5'-CCATGGTAAGAAAAGGAGAAAAAAAATTAGC-3') and Sal-TpsbA-Rv (5'-GTCGACCGAATATAGCTCTTCTTTCTTATT-3'). The amplification program using primer pairs 1 and 2 was as follows: 94°C for 1 min, followed by 30 cycles at 94°C for 30 s, 53°C for 30 s, and 72°C for 2 min. The transgenic lines confirmed by genotyping were subjected to two more rounds of regeneration cycles on RMOP agar medium containing 500 mg/L spectinomycin dihydrochloride to achieve homoplasmy and finally rooted on MS medium supplemented with 3% (w/v) sucrose, 0.8% (w/v) agar, and 500 mg/L spectinomycin dihydrochloride.

### Southern Blot Analysis

Total cellular DNA was extracted from wild-type and transplastomic tobacco leaves using the DNeasy Plant Mini Kit (QIAGEN, Valencia, CA, United States) according to the manufacturer’s instructions. One microgram of total cellular DNA was digested with *Xmn*I, separated by 1% (w/v) agarose gel electrophoresis, and transferred to an Amersham Hybond-N^+^ positively charged nylon membrane (GE Healthcare, Chicago, IL, United States). The DNA probe covering a part of the tobacco plastid *rrn16*-*rrn23* operon was generated using the PCR DIG Probe Synthesis Kit (Roche/Merck KGaA, Darmstadt, Germany) with wild-type tobacco total cellular DNA as a template and a primer pair: ADL-F2 (5'-CCCCTTTTTTACGTCCCCATGTTCC-3') and ADL-R2 (5'-GCCTTTCCTCGTTTGAACCTCGCCC-3'). The amplification program was as follows: 95°C for 2 min, followed by 30 cycles at 95°C for 30 s, 58°C for 30 s, 72°C for 1 min, and 72°C for 7 min. Hybridization was performed overnight at 60°C using DIG Easy Hyb (Roche/Merck KGaA, Darmstadt, Germany). The hybridized membrane was then washed, and the probe DNA was detected using a DIG Luminescent Detection Kit (Roche/Merck KGaA, Darmstadt, Germany) according to the manufacturer’s instructions. Immunodetectable bands on the membrane were visualized using the Chemiluminescence Imaging System, FUSION SL4 (Vilber-Lourmat, France).

### SDS-PAGE and Western Blot Analysis

To extract total cellular proteins from leaves of wild-type and transplastomic tobacco plants grown on MS medium or in soil, leaf material (200 mg) was homogenized in 1 ml of pre-chilled phosphate-buffered saline (PBS) (pH 7.4). The homogenate was centrifuged at 20,000 × *g* for 5 min at 4°C, and the supernatant was collected as a soluble fraction. The resulting pellet was resuspended in an equal volume of PBS and 2× Laemmli Sample Buffer (Bio-Rad, Hercules, CA, United States), and centrifuged at 20,000 × *g* for 5 min at 4°C. The resulting supernatant was collected as the insoluble fraction. The protein concentration in the soluble fraction (total soluble protein; TSP) was quantified using BCA Protein Assay Kit (TaKaRa Bio Inc., Kusatsu, Japan) according to the manufacturer’s instructions. The protein extracts were separated by 12.5% or 13% sodium dodecyl sulfate-polyacrylamide gel electrophoresis (SDS-PAGE) and subjected to Coomassie Brilliant Blue (CBB) staining or Western blot analysis. For Western blot analysis, proteins were transferred to polyvinylidene difluoride (PVDF) membranes (Hybond P; GE Healthcare, Chicago, IL, United States). After blocking, the blots were incubated for 1 h with a custom-made rabbit antiserum against a mixture of two synthetic peptides derived from amino acid residues 170-180 and 209-220 of RGNNV-CP, purchased from Eurofins Genomics (Tokyo, Japan), and then washed with T-PBS [PBS containing 0.1% (w/v) Tween-20]. The blots were then incubated with anti-rabbit IgG and HRP-linked antibody (Cell Signaling Technology, Danvers, MA, United States) at a dilution of 1:20,000 for 1 h. After washing the membranes with T-PBS, immunoblot detection was performed using a Chemiluminescence Imaging System (FUSION SL4, Vilber-Lourmat, France) and ECL Prime Western blotting detection reagent (GE Healthcare, Chicago, IL, United States). The expression levels of RGNNV-CP were quantified by densitometric analysis using Fusion-Capt software (Vilber Lourmat, France).

### Expression and Purification of RGNNV-VLPs in *Escherichia coli*

DNA fragments flanked by *Nco*I and *Not*I sites encoding the codon-optimized *RGNNV-CP* for expression in *E. coli* (*RGNNV-CPec*) or its variant with a 6×His tag at the C-terminus (*RGNNV-CPec-(GGGS)_3_-His_6_*) were synthesized by Eurofins Genomics (Tokyo, Japan). Synthetic DNA fragments (*RGNNV-CPec* and *RGNNV-CPec-(GGGS)_3_-His_6_*) were inserted into pET-28a (Novagen; Merck KGaA, Darmstadt, Germany) digested with *Nco*I and *Not*I to obtain pET-RGNNV and pET-RGNNV-His, respectively.

The pET-RGNNV or pET-RGNNV-His was expressed in *E. coli* BL21 (DE3), and the cells were cultured in Luria-Bertani (LB) broth containing 20 mg/ml kanamycin at 37°C until the OD at 600 nm reached 0.3–0.6. To induce gene expression, isopropyl β-D-thiogalactopyranoside (IPTG) was added to a final concentration of 1 mM, and the cells were cultured at 30°C for 2 h. Cells were harvested and disrupted in PBS using a sonicator (Sonifier SFX250, Branson, Danbury, CT, United States). After centrifugation, the resulting supernatant was layered on a 30% (w/w) sucrose cushion and ultracentrifuged at 250,000 × *g* for 60 min at 4°C using a Beckman TLS-55 swinging bucket rotor (Beckman Coulter Inc., Brea, CA, United States). The resulting pellet was resuspended in PBS and then layered onto a 10–40% (w/w) discontinuous sucrose gradient and ultracentrifuged at 250,000 × *g* for 20 min at 4°C using a Beckman TLS-55 swinging bucket rotor. The collected fractions were analyzed by SDS-PAGE to confirm the fractions enriched with RGNNV-VLPs. The desired fractions containing RGNNV-VLPs were diafiltrated and concentrated using the Amicon Ultra-15 Centrifugal Filter Unit [10,000 molecular weight cutoff (MWCO); Merck KGaA, Darmstadt, Germany], according to the manufacturer’s instructions.

### Purification of Plant-Derived RGNNV-VLPs

Mature leaves (200 mg leaf fresh weight) from transplastomic tobacco plants were homogenized in 1 ml of pre-chilled PBS (pH 7.4). After centrifugation at 20,000 × *g* for 20 min at 4°C, the resulting supernatant was layered onto a 10–40% (w/w) discontinuous sucrose gradient and centrifuged at 141,000 × *g* for 3 h at 4°C using a Beckman SW28 swinging bucket rotor (Beckman Coulter Inc., Brea, CA, United States). The collected fractions were analyzed by SDS-PAGE to confirm the fractions enriched with RGNNV-VLPs. The desired fractions containing RGNNV-VLPs were diafiltrated and concentrated using the Amicon Ultra-15 Centrifugal Filter Unit (10,000 MWCO; Merck KGaA, Darmstadt, Germany), according to the manufacturer’s instructions.

### Transmission Electron Microscopy (TEM)

For negative staining, RGNNV-VLPs purified from *E. coli* or tobacco plants were diluted to 0.8 mg/ml, placed on freshly glow-discharged copper grids coated with carbon (Excel support film, 200 mesh, Nisshin EM Co., Tokyo, Japan), and allowed to dry completely. The grids were rinsed with water, negatively stained with 2% (w/v) uranyl acetate, and then dried. Micrographs were obtained using a transmission electron microscope (JEOL JEM-1400, Tokyo, Japan) operating at 80 kV. Digital images were captured using a charge-coupled device (CCD) camera (EM-14321DCAM; Hamamatsu Photonics K. K., Hamamatsu, Japan).

For ultrastructural observations, mature tobacco leaf samples were fixed with 2% paraformaldehyde (PFA) and 2% glutaraldehyde (GA) in 0.05 M cacodylate buffer (pH 7.4) overnight at 4°C. After fixation, the samples were washed with 0.05 M cacodylate buffer and post-fixed with 2% OsO4 in 0.05 M cacodylate buffer for 3 h. The samples were then dehydrated overnight in a series of graded ethanol solutions, infiltrated with propylene oxide (PO), placed in a 50:50 mixture of PO and resin (Quetol-651; Nisshin EM Co., Tokyo, Japan) for 3 h, then transferred to a 100% resin, and polymerized at 60°C for 48 h. The polymerized resins were cut into 80-nm ultrathin sections with a diamond knife using an ultramicrotome (Ultracut UCT; Leica, Vienna, Austria), and the sections were mounted on copper grids and counterstained with 2% uranyl acetate and Lead stain solution (Sigma-Aldrich Co., Tokyo, Japan). The grids were observed using a transmission electron microscope (JEM-1400Plus; JEOL Ltd., Tokyo, Japan) at an acceleration voltage of 100 kV. Digital images were captured using a CCD camera (EM-14830RUBY2; JEOL Ltd., Tokyo, Japan).

### Fish

Hatchery-reared young sevenband grouper (*Epinephelus septemfasciatus*) with an average body weight of 25.8 g were immunized intraperitoneally and orally with crudely purified protein extracts from tobacco mature leaves. Fish were reared at approximately 25°C in 1 m^3^ tanks with a flow-through system, using water previously disinfected with ultraviolet (UV) light. All animal experiments were conducted in accordance with “Fundamental Guidelines for Proper Conduct of Animal Experiment and Related Activities in Academic Research Institutions under the jurisdiction of the Ministry of Education, Culture, Sports, Science and Technology (Notice No. 711)” and approved by the Ehime Fisheries Research Center. Prior to immunization experiments, 20 fish from the fish stock were randomly sampled and their brains were subjected to reverse transcriptase-polymerase chain reaction (RT-PCR) test for detecting betanodavirus as described by Nishioka et al. (2016). No positive PCR products were detected in any of the examined fish.

### Virus

The SGEhi00 strain of RGNNV genotype (Yamashita et al., 2005) was propagated in E-11 cells in Leibovitz’s L-15 medium (Thermo Fisher Scientific, Waltham, MA, United States) supplemented with 5% fetal bovine serum (FBS) (Thermo Fisher Scientific, Waltham, MA, United States) at 25°C. Viral infectivity titers were determined and stored at −80°C until use, as described by Iwamoto et al. (2000).

### Immunization of Fish

Sevenband groupers were immunized by intraperitoneal injection or oral administration of crudely purified protein extracts from wild-type and transplastomic tobacco leaves. Mature leaves were homogenized in pre-chilled PBS and centrifuged at 20,000 × *g* for 20 min at 4°C. The resulting supernatants were filtered using a filter (Millex-GP Syringe Filter Unit, 0.22 μm, polyethersulfone, 33 mm, gamma sterilized; Merck KGaA, Darmstadt, Germany) to remove cell debris. The filtrate was diafiltrated with PBS using an Amicon Ultra-15 Centrifugal Filter Unit (10,000 MWCO; Merck KGaA, Darmstadt, Germany) to reduce the contamination of alkaloids such as nicotine. The concentration of recovered proteins was quantified using BCA Protein Assay Kit (TaKaRa Bio Inc., Kusatsu, Japan), and the protein samples were diluted to 2 mg/ml in PBS and stored at −80°C until use.

A total of 280 fish were randomly divided into seven groups (approximately 40 fish per group). For parenteral vaccination, fish were immunized intraperitoneally with crudely purified protein extracts (200 μg/fish) from wild-type or transplastomic tobacco leaves, a commercial formalin-inactivated virus vaccine (OceanTect VNN; Nisseiken Co., Ltd., Ome, Japan) as positive control, or PBS as negative control. For oral vaccination, fish were fed commercial diets supplemented with crudely purified protein extracts from wild-type or transgenic tobacco leaves (200 μg/fish) or PBS (0.1 ml/fish) as a negative control, once a day for five consecutive days. After immunization, the experimental fish were transferred to 45 L plastic tanks at 25°C (±1.0°C), supplied with UV-treated water, and fed with commercial pellets once a day. On day 21 after immunization, blood samples were collected from the caudal veins of 8–10 fish per group to measure anti-RGNNV neutralizing antibody levels, and the remaining fish were subjected to a viral challenge test.

### Viral Challenge Test

Fish were intramuscularly challenged with RGNNV (SGEhi00 strain) at a dose of 10^3.3^ TCID_50_ per fish. The experimental fish were reared in 45 L tanks at 25°C (±1.0°C) for 14 days after challenging with virus and monitored for mortality. The relative percent survival (RPS; Amend, 1981) was calculated from the cumulative mortality using the following formula:

$$RPS=\left\{1-\left(\frac{\%mortalityofexperimentalgroup}{\%mortalityofcontrolgroup}\right)\right\}\times 100$$

### Virus-Neutralizing Antibody Assay

Anti-RGNNV neutralizing antibody titers in sera were measured using E-11 cells and SGEhi00 strains, as described by Yamashita et al. (2005). The detection limit for antibody titer was 1:80.

### Statistical Analysis

The cumulative mortality and morbidity of fish after the RGNNV challenge were analyzed using Fisher’s exact probability test. The levels of anti-RGNNV neutralizing antibodies between the vaccinated and PBS-control fish groups were tested by Student’s *t*-test. Statistical significance was set at *p* < 0.05.

## Results

### Construction of Plastid Transformation Vector and Generation of Transplastomic Tobacco Plants

**Figure 1:**
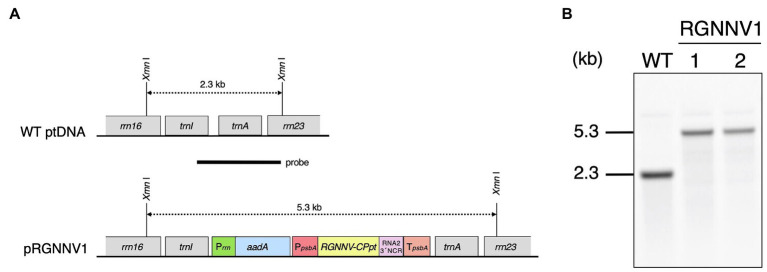
Generation of transplastomic tobacco plants expressing RGNNV capsid protein (RGNNV-CP). **(A)** Physical map of the target region in the plastid genome (WT ptDNA) and schematic diagram of the chloroplast transformation vector, pRGNNV1 (construct not drawn to scale). Synthetic DNA covering the codon-optimized *RGNNV-CP* (*RGNNV-CPpt*) and the subsequent 3' non-cooding regioon (3' NCR) derived from the RNA2 genome of red-spotted grouper NNV (RGNNV) is driven by the tobacco plastid *psbA* promoter and its 5'-UTR (P*psbA*), and the transcript is designed to be stabilized by the 3'-UTR of tobacco plastid *psbA* (T*psbA*). The selectable marker gene *aadA* is under the control of the tobacco plastid ribosomal RNA operon promoter (P*rrn*). The transgenes are targeted to the intergenic spacer region between *trnI* and *trnA* in the tobacco plastid genome. The location of the probe used in Southern blot analysis is shown as a black bar. The wild-type and the transgenic chloroplast genomes give rise to hybridization signals corresponding to 2.3 kb and 5.3 kb *Xmn*I-*Xmn*I DNA fragments, respectively. **(B)** Southern blot analysis of two independent transgenic lines (RGNNV1-1 and RGNNV1-2). Total cellular DNA was digested with *Xmn*I and subjected to hybridization analysis with a DIG-labeled probe shown in **(A)**.

The plastid transformation vector pRGNNV1 was constructed to transform the chloroplast genomes. This vector contains a synthetic *RGNNV-CP* gene (*RGNNV-CPpt*) with codon usage optimized for expression in plastids. The coding region of *RGNNV-CPpt* was designed to be followed by a 3' non-coding region (3' NCR) derived from the RNA2 genome of RGNNV. The 3' NCR has been reported to be required for high expression of RGNNV-CP in avian cell lines (Huang et al., 2007). To further increase the expression level of *RGNNV-CPpt*, the transgene was driven by a promoter derived from tobacco plastid *psbA* and its subsequent 5'-untranslated region (5'-UTR), and the transcript was stabilized by the 3'-UTR of tobacco plastid *psbA*. This expression cassette was inserted into the *trnI-trnA* intergenic region derived from the tobacco plastid DNA on the universal plastid transformation vector p16S-aadA-23S(T) with the spectinomycin resistance gene, *aadA*, as a selectable marker, to obtain pRGNNV1 (Figure 1A).

Plastid transformation was carried out by the biolistic method using pRGNNV1, and 36 bombardments resulted in 11 independent spectinomycin-resistant shoots/calli. Shoots regenerated after the second round of selection with spectinomycin were genotyped by PCR using a primer pair (ADL-F3 and ADL-R3) to infer site-specific incorporation of the transgene and another primer pair (RGNNV-Fd1 and Sal-TpsbA) to confirm the presence of the transgene. The results demonstrated that three of the regenerating shoots were PCR-positive, and that six were PCR-negative and considered to be spontaneous mutants (data not shown). For the remaining two regenerating shoots, the presence of the transgene was confirmed, but it was not targeted to the *trnI*-*trnA* intergenic region (data not shown). In both the shoots, the transgene may have been mis-targeted due to homologous recombination *via* the *psbA* promoter and/or the 5'-, 3'-UTR of *psbA* used in the expression cassette. After the three positive shoots were subjected to two more rounds of regeneration cycles on RMOP agar medium containing 500 mg/L spectinomycin dihydrochloride to achieve homoplasmy, they were finally rooted on MS agar medium supplemented with 500 mg/L spectinomycin dihydrochloride. Two of the three lines showed a normal phenotype, but one line exhibited abnormal leaf morphology with rounding and unevenness, probably due to somatic mutation caused by tissue culture. Therefore, two independent transformants with normal phenotypes (RGNNV1-1 and RGNNV1-2) were subjected to further analysis.

To verify the site-specific integration into the plastid genome and to confirm homoplasmy, Southern blot analysis was performed using a probe covering the flanking region (Figure 1A). The expected size of DNA fragments digested with *Xmn*I was 2.3 kb and 5.3 kb for wild-type and the transformed plastid DNA, respectively (Figure 1A). There was no detectable 2.3 kb band in the two transgenic lines, indicating that all copies of the plastid genome were transformed (Figure 1B). The resulting homoplasmic T_0_ lines were grown in soil for seed collection, and T_1_ generation plants were used for all subsequent analyses.

### High-Level Expression of RGNNV-CP in Transplastomic Tobacco Plants

**Figure 2:**
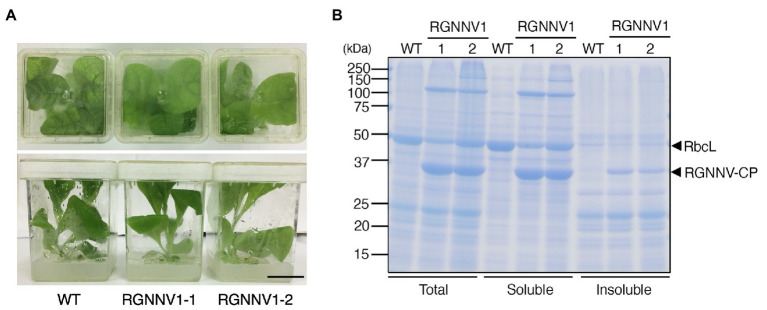
Transplastomic plants expressing RGNNV-CP grown under photoheterotrophic conditions. **(A)** Phenotype of wild-type (WT) and transplastomic (RGNNV1-1 and RGNNV1-2) plants grown on MS agar medium containing 3% (w/v) sucrose as carbon source. Bar = 3 cm. **(B)** Detection of RGNNV-CP accumulated in transplastomic plants (RGNNV1-1 and RGNNV1-2) by Coomassie Brilliant Blue (CBB) staining. Total, soluble, and insoluble protein extracts from 1.5 mg of fresh leaf were separated by 12.5% sodium dodecyl sulfate-polyacrylamide gel electrophoresis (SDS-PAGE) gel and stained with CBB. The 37-kDa and 55-kDa bands correspond to RGNNV-CP and large subunit of Rubisco (RbcL), respectively.

When grown on MS agar medium supplemented with sucrose as a carbon source, the two transplastomic lines (RGNNV1-1 and RGNNV1-2) grew normally and showed a visible phenotype similar to that of the wild-type plants (Figure 2A). Protein extracts from mature leaves of wild-type and transgenic plants grown on the synthetic medium were subjected to SDS-PAGE followed by CBB staining. Protein extracts from the transgenic plants showed a specific band of approximately 37 kDa, which is expected for RGNNV-CP, mainly as a soluble protein, and its accumulation level was higher than that of the large subunit of Rubisco (RbcL) (Figure 2B).

**Figure 3:**
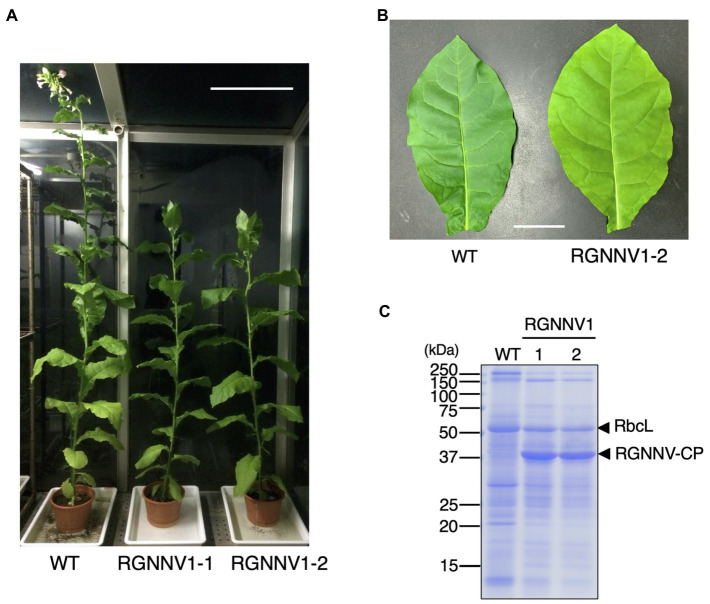
Transplastomic plants expressing RGNNV-CP grown under photoautotrophic conditions. **(A)** Soil-grown wild-type (WT) and transplastomic (RGNNV1-1 and RGNNV1-2) plants were cultivated in a phytotron using sunlight and natural daylength, with a temperature cycle of 12 h at 26°C and 12 h at 23°C. Bar = 30 cm. **(B)** Mature leaves of WT and RGNNV1-2 plants. Bar = 5 cm. **(C)** Detection of RGNNV-CP accumulated in transplastomic plants (RGNNV1-1 and RGNNV1-2) by CBB staining. Total soluble protein (TSP) extracted from 1.5 mg of fresh leaf was separated by 13% SDS-PAGE gel and stained with CBB.

**Figure 4:**
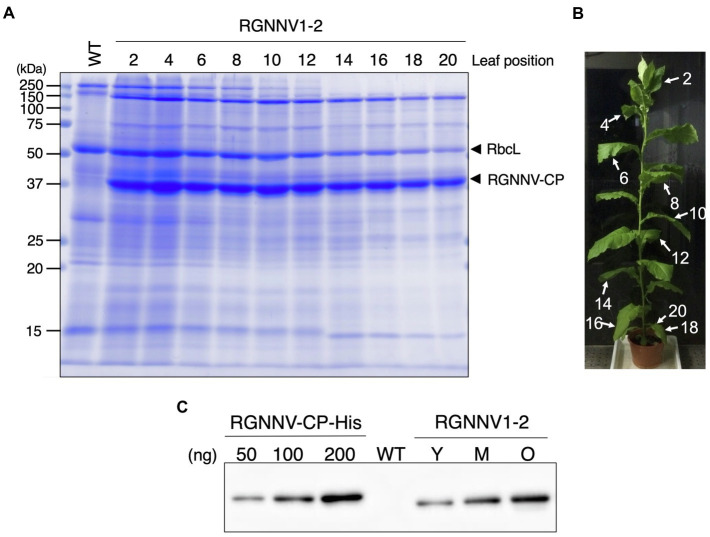
High accumulation of RGNNV-CP in all leaves of the transgenic tobacco plant. **(A)** TSPs extracted from 1.5 mg of different leaves of a transplastomic plant (RGNNV1-2) were separated by 13% SDS-PAGE and stained with CBB. TSP extracted from a mature leaf of wild-type (WT) plant was loaded as a control. **(B)** The RGNNV1-2 plant used for the analysis shown in **(A)**. The leaves were numbered from top to bottom. **(C)** Western blot analysis to detect RGNNV-CP accumulated in the transplastomic plant. TSPs (500 ng) extracted from young (Y), mature (M), and old senescent (O) leaves of RGNNV1-2 correspond to leaf nos. 2, 10, and 20 in **(B)**, respectively, were analyzed. TSP (5,000 ng) extracted from a mature leaf of wild-type (WT) was also loaded as a control. Blots were detected using an antiserum against a mixture of synthetic peptides derived from RGNNV-CP. A dilution series (50, 100, and 200 ng) of purified 6×His-tagged RGNNV-CP (RGNNV-CP-His) expressed in *Escherichia coli* was analyzed as a standard.

When the transplastomic plants were grown in soil, they showed a weak pale green phenotype (Figure 3B) and significant growth retardation compared to wild-type plants (Figure 3A), but eventually grew to the same height as the wild-type plants, and viable seeds were obtained. Total soluble protein (TSP) extracted from mature leaves of soil-grown plants was analyzed by SDS-PAGE followed by CBB staining. As a result, a prominent band of approximately 37 kDa, corresponding to the expected molecular weight of RGNNV-CP, was specifically detected in the extracts of two independent transgenic lines (RGNNV1-1 and RGNNV1-2), which was not found in the wild-type plant extracts (Figure 3C). Since the biosynthetic capacity of proteins decreases with leaf age, we evaluated the stability of RGNNV-CP by examining the RGNNV-CP accumulation in each leaf. The results showed that the accumulation of most proteins, including RbcL, gradually decreased with senescence, while RGNNV-CP was highly expressed in young leaves and stably accumulated in mature and old senescent leaves (Figure 4A). To further confirm that the 37-kDa band was derived from *RGNNV-CPpt*, Western blot analysis was performed using an antiserum against a mixture of two synthetic peptides derived from RGNNV-CP. The results showed that the 37-kDa protein in transplastomic plants was identical to the gene product of *RGNNV-CPpt*, and no signal was obtained in wild-type plants (Figure 4C). The accumulation level of RGNNV-CP was quantified by Western blot analysis using a dilution series of the His-tagged RGNNV-CP recombinant protein expressed and purified in *E. coli* as a standard. Three independent experiments showed that RGNNV-CP accounted for 10.2, 20.1, and 22.7% of TSP in young, mature, and old senescent leaves of the transgenic plants, respectively (Figure 4C). Based on the protein content extracted from the leaves, the production yield of RGNNV-CP was estimated to be 3.5, 3.4, and 2.3 mg/g leaf fresh weight in young, mature, and old senescent leaves of the transplastomic plants, respectively.

### RGNNV-CP Efficiently Self-Assembles Into VLPs in Chloroplast Stroma

**Figure 5:**
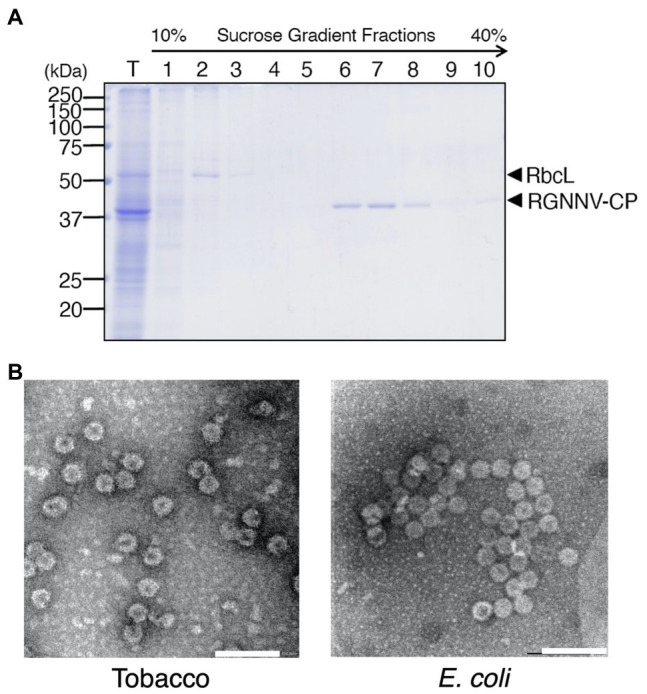
Purification of RGNNV virus-like particles (RGNNV-VLPs) from transplastomic tobacco plants. **(A)** TSP (T) extracted from mature leaves of transplastomic plants (RGNNV1-2) was analyzed by sucrose gradient sedimentation, and RGNNV-CP in each fraction was detected by CBB staining. Fraction 1 was obtained from the top of the tube. **(B)** Electron micrograph of the RGNNV-CP-enriched fractions recovered by the sucrose gradient sedimentation analysis shown in **(A)**. RGNNV-VLPs derived from the transplastomic tobacco plants (Tobacco) and *E. coli* were visualized by negative staining with 2% (w/v) uranyl acetate and transmission electron microscopy (TEM). Bar = 100 nm.

RGNNV-CP was expressed in large amounts in the transplastomic plants and showed high stability even in old senescent leaves, suggesting that the recombinant protein was present in tobacco chloroplasts as VLPs. To confirm this, TSP extracted from the transgenic plants was analyzed by sucrose sedimentation. Among the 10 fractions recovered (fraction 1 obtained from the top of the tube), Rubisco was enriched in fractions 2 and 3, whereas RGNNV-CP was detected in fractions 6–10 with higher molecular weight (Figure 5A). This result is consistent with the fact that the Rubisco holoenzyme in tobacco plants is a 550 kDa RbcL8RbcS8 form (Wostrikoff and Stern, 2007), whereas 180 copies of RGNNV-CP arranged with *T* = 3 symmetry self-assemble into a 6,660-kDa VLP (Bandín and Souto, 2020). When fractions enriched with RGNNV-CP were collected and analyzed by transmission electron microscopy (TEM), spherical particles with a diameter of approximately 25–30 nm were frequently observed (Figure 5B). The morphology and size of the chloroplast-derived RGNNV-VLPs were similar to those expressed in *E. coli* (Figure 5B).

**Figure 6:**
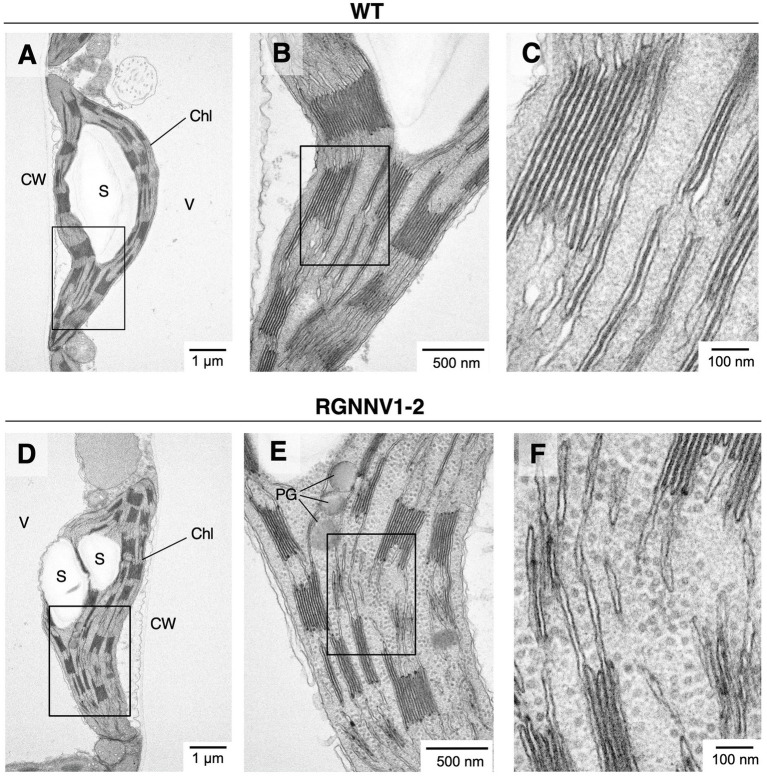
Electron micrographs of RGNNV-VLPs present in high density in chloroplast stroma of transplastomic tobacco plants. *In situ* TEM images of chloroplasts from mature leaves of wild-type [WT; **(A–C)**] and the transplastomic [RGNNV1-2; **(D–F)**] plants. **(B)**, **(C)**, **(E)**, and **(F)** are magnified views of the box regions in **(A)**, **(B)**, **(D)**, and **(E)**, respectively. Chl, chloroplast; S, starch granule; V, vacuole; CW, cell wall; and PG, plastoglobule.

To investigate whether RGNNV-VLPs are formed in tobacco chloroplasts, *in situ* TEM observations were performed. Although the grana stacked in the chloroplasts of the transformants tended to be slightly smaller than those of wild-type tobacco, there were no significant differences in chloroplast size or morphology (Figure 6). Interestingly, however, the chloroplast stroma of the transplastomic plants contained uniformly dispersed spherical particles with a diameter of approximately 25–30 nm (Figure 6). Such structures were not observed in the chloroplasts of the wild-type plants (Figure 6). These results indicate that RGNNV-CP can self-assemble into VLPs in tobacco chloroplasts.

### Chloroplast-Derived RGNNV-VLPs Are Highly Immunogenic Against RGNNV Challenge

To investigate the immunogenicity of chloroplast-derived RGNNV-VLPs, we planned to conduct a viral challenge test after intraperitoneal injection and oral administration of VLPs to the fish (sevenband grouper). Although it is convenient to use crude TSP extracts containing chloroplast-derived RGNNV-VLPs or lyophilized powder of the transgenic tobacco leaves as vaccine candidates, there are concerns that alkaloid, such as nicotine, contained in tobacco leaves may adversely affect fish or reduce the immunogenicity of chloroplast-derived RGNNV-VLPs. In fact, when *Staphylococcus aureus*-derived antigenic protein (Efb) was expressed in tobacco and lyophilized powder of transgenic leaves was orally administered to mice, transgenic leaves of a low-alkaloid tobacco variety (cv. 81V9) showed significantly higher immunogenicity than the transgenic leaves of a common tobacco variety (cv. Samsun) (Festa et al., 2013). Therefore, ultrafiltration was employed to reduce the alkaloid content of TSP extracted from the wild-type and transplastomic tobacco leaves. Although other methods have been reported to be more effective in removing nicotine from crude protein extracts derived from tobacco leaves (Fu et al., 2010), ultrafiltration using a centrifugal filter device was chosen because it is a simple method and is believed to have less effect on the VLP structure during the purification process. Comparison of the protein composition of TSPs extracted from the leaves of wild-type and transgenic plants before and after the ultrafiltration treatment showed no noticeable differences (Supplementary Figure S1). In a preliminary experiment, ultrafiltered protein extracts (200 μg/fish) from wild-type and transplastomic leaves were administered intraperitoneally or orally to sevenband grouper, and no lethal symptoms, abnormal swimming behavior, or reduced growth rate were observed, suggesting that the level of alkaloids remaining in the protein extracts after ultrafiltration was not considered to be harmful to fish.

**Figure 7:**
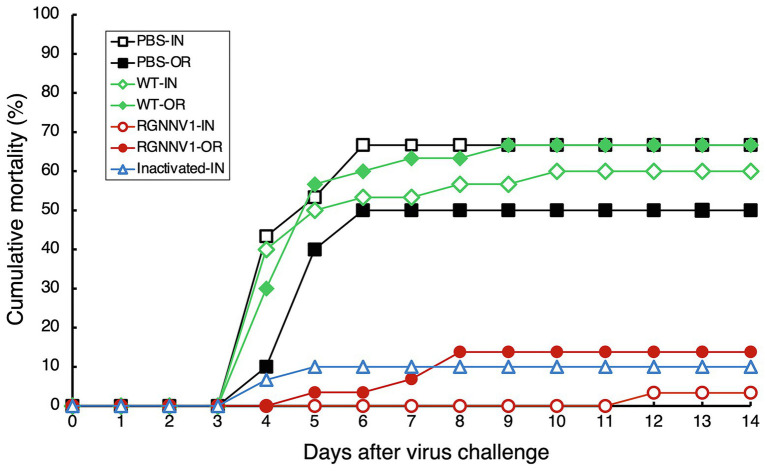
Cumulative mortality after RGNNV challenge in sevenband grouper immunized with chloroplast-derived RGNNV-VLPs. Fish were vaccinated with PBS as control, crudely purified protein extracts from mature leaves of wild-type (WT) or the transplastomic (RGNNV1) tobacco plants, or a commercial inactivated vaccine (Inactivated) by intraperitoneal injection (IN) or oral administration (OR). On day 21 after immunization, the fish were challenged by intramuscular injection with RGNNV at a dose of 10^3.3^ TCID_50_ per fish and observed for an additional 14 days.

For injection vaccination, ultrafiltered protein extracts from the transplastomic leaves (200 μg/fish; approximately equivalent to 1.5 μg/fish body weight of RGNNV-CP), the same amount of protein extracts from wild-type leaves, a commercially available formalin-inactivated vaccine, or PBS as a negative control were administrated intraperitoneally to the fish without adjuvant. Twenty-one days post-immunization, the experimental fish were challenged with RGNNV, and mortality was monitored. The results showed that the cumulative mortality of fish immunized with PBS and wild-type tobacco-derived protein extracts was 66.7 and 60.0%, respectively. In contrast, the cumulative mortality of fish groups vaccinated with the commercial vaccine and protein extracts containing chloroplast-derived RGNNV-VLPs was only 10.0 and 3.3%, respectively (Figure 7). This indicates that the RPS (Amend, 1981) of the fish injected intraperitoneally with the commercial vaccine and the protein extracts containing chloroplast-derived RGNNV-VLPs was 85.0 and 95.1%, respectively, revealing that the chloroplast-derived RGNNV-VLPs were more immunogenic than the commercial inactivated vaccine (Table 1).

**Table 1:** Immunogenicity of chloroplast-derived RGNNV-VLPs against RGNNV-challenge in sevenband grouper fish.

Fish group^a^	Cumulative mortality (%)[No. of dead/No. of challenged]	RPS^b^ (%)	Cumulative morbidity (%)[(No. of dead + No. of showing abnormal swimming)/No. of challenged]
PBS-IN	66.7 [20/30]		66.7 [(20 + 0)/30]
WT-IN	60.0 [18/30]	10.0	70.0 [(18 + 3)/30]
RGNNV1-IN	3.3 [1/30]^*^	95.1	3.3 [(1 + 0)/30]^*^
Inactivated-IN	10.0 [3/30]^*^	85.0	20.0 [(3 + 3)/30]^*^
PBS-OR	50.0 [15/30]		73.3 [(15 + 7)/30]
WT-OR	66.7 [20/30]	−33.4	73.3 [(20 + 2)/30]
RGNNV1-OR	13.8 [4/29]^*^	72.4	13.8 [(4 + 0)/29]^*^

a Fish were vaccinated with PBS as a control, crudely purified protein extracts from mature leaves of wild-type (WT) or transplastomic (RGNNV1) tobacco plants, or a commercial inactivated vaccine (Inactivated) by intraperitoneal injection (IN) or oral administration (OR). On day 21 after immunization, the fish were challenged by intramuscular injection with RGNNV at a dose of 10^3.3^ TCID_50_ per fish and observed for an additional 14 days.

b RPS, Relative percent survival.

* *p* < 0.01.

For oral vaccination, ultrafiltered protein extracts from the transplastomic leaves (200 μg/fish; approximately equivalent to 1.5 μg/fish body weight of RGNNV-CP), the same amount of protein extracts from wild-type leaves, or PBS were mixed with commercial feed without adjuvant and fed to the fish once a day for five consecutive days. This oral vaccination relied on the fish to voluntarily eat the feed, making it difficult to strictly control the vaccine dosage. However, it is ideal for use at the practical application stage, and the same method has been used to investigate the immunogenicity of *E. coli*-derived OSGNNV-VLPs (Chien et al., 2018) and yeast-derived RGNNV-VLPs (Cho et al., 2017). A commercially available inactivated vaccine was not used in this experiment because it has been reported that it does not show significant immunogenicity when administered orally without adjuvant (Gaafar et al., 2018). As in the case of parenteral vaccination, the experimental fish were challenged with RGNNV 21 days after immunization and then monitored for mortality. The results showed that the cumulative mortality of the fish immunized with PBS and wild-type tobacco-derived protein extracts reached 50 and 66.7%, respectively (Figure 7). In contrast, the cumulative mortality of the fish group immunized with the protein extracts containing chloroplast-derived RGNNV-VLPs was only 13.8% (Figure 7), and the RPS was calculated to be 72.4% (Table 1). It seems counterintuitive that the cumulative mortality of the fish group immunized with wild-type tobacco-derived protein extracts (66.7%) was higher than that of the fish group immunized with PBS (50%), but the difference was not statistically significant (Table 1). Furthermore, when the cumulative morbidity was calculated by adding the number of surviving fish showing symptoms of the disease (abnormal swimming) to the number of dead fish, the morbidity of both fish groups immunized with PBS and wild-type tobacco-derived protein extracts was the same at 73.3% (Table 1). Therefore, the above results were considered to be due to experimental error and not due to toxic factors other than nicotine. Compared with the cumulative morbidity of the fish group vaccinated with PBS and with wild-type tobacco-derived protein extracts, the cumulative morbidity of the fish vaccinated with the protein extracts containing chloroplast-derived RGNNV-VLPs was significantly lower (13.8%) (Table 1), indicating that chloroplast-derived RGNNV-VLPs are immunogenic even when administered orally.

Serum was collected from each group of immunized fish, and the titer of anti-RGNNV neutralizing antibody was measured. The results showed that intraperitoneal injection of protein extracts containing chloroplast-derived RGNNV-VLPs elicited a high level of neutralizing antibodies (1:2600 ± 1913.1; *p* < 0.01). Statistically significant induction of neutralizing antibodies was also observed when the same protein extracts were administered orally (1:138 ± 49.1; *p* < 0.01) and when the commercial inactivated vaccine was administered by injection (1:160 ± 106.5; *p* < 0.05) (Supplementary Figure S2). This result is in agreement with the results of the RGNNV challenge test (Figure 7).

## Discussion

In this study, transgenic tobacco plants producing RGNNV-VLPs, a promising vaccine candidate against VNN, were developed using chloroplast genetic engineering. Consequently, high levels of RGNNV-CP accumulation (averaging about 3 mg/g leaf fresh weight) were achieved in all young, mature, and old senescent leaves (Figure 4). This may be due to the optimization of codon usage of *RGNNV-CP* for expression in chloroplasts, the use of the *psbA* promoter and its 5'-UTR, which are responsible for the high transcriptional and translational activity, respectively, and the utilization of the *psbA* 3'-UTR, which helps stabilize the transcript. A number of reports described successful high expression of transgenes using the *psbA*-derived gene expression cassette (Cardi et al., 2010; Jin and Daniell, 2015). In addition, among the several reports on chloroplast expression of the HPV-16 L1 capsid protein (Fernández-San Millán et al., 2008; Lenzi et al., 2008; Waheed et al., 2011), the highest expression level (24% of TSP) was achieved using a *psbA*-derived gene expression cassette (Fernández-San Millán et al., 2008). However, it is unlikely that the 3' NCR derived from RNA2 of RGNNV, which has been reported to contribute to the high expression of RGNNV-CP in avian cell lines (Huang et al., 2007), is also involved in the high accumulation of RGNNV-CP in chloroplasts. This is because we produced transplastomic tobacco plants expressing RGNNV-CP without the 3' NCR of RNA2 and observed that the accumulation level of RGNNV-CP was comparable to those of RGNNV1-1 and RGNNV1-2 with the 3' NCR (data not shown). Another reason for the high accumulation of RGNNV-CP in all leaves of the transplastomic tobacco plants may be that RGNNV-CP self-assembles into VLPs in the chloroplast stroma with high efficiency (Figures 5, 6), and the resulting higher-order molecular structures are resistant to proteolysis in chloroplasts. In this study, we investigated the accumulation level of RGNNV-CP in the T_1_ generation plants. Since one of the advantages of plastid transformation is the absence of gene silencing, as seen in nuclear transformants, high expression levels are likely to be maintained in successive generations. Nevertheless, actual confirmation of the above is essential for future practical applications.

The expression levels of RGNNV-CP in the transplastomic tobacco plants generated in this study (averaging about 3 mg/g leaf fresh weight) are more than 100-fold higher compared to the productivity of ACNNV-VLPs transiently expressed in tobacco leaves (10 mg/kg leaf fresh weight) in a previous study (Marsian et al., 2019). Prior to this study, the expression of NNV-CP using chloroplast genetic engineering in tobacco has been reported, where, unlike our transformants, *NNV-CP* was transcribed by the rice plastid-derived *clpP* promoter, and the accumulation of NNV-CP in transplastomic tobacco plants was confirmed by Western blot analysis, however, quantitative analysis of expression levels has not been performed (Cho et al., 2018). Although a simple comparison cannot be made, it is likely that the transplastomic plants developed in this study, which expressed *RGNNV-CP* using the *psbA*-derived gene expression cassette, accumulated higher levels of the target capsid protein than the transgenic plants in the previous study. One rationale for this is that the *psbA* promoter shows much higher transcriptional activity than the *clpP* promoter in chloroplasts (Legen et al., 2002). Furthermore, the yield of RGNNV-CP produced by the transplastomic tobacco plants in this study (averaging approximately 3 g/kg leaf fresh weight) was comparable to or better than that of 100 mg of OSGNNV-VLPs purified from 1 L of *E. coli* culture (Chien et al., 2018) and 60 mg of RGNNV-CP expressed in 1 L of yeast culture (Jeong et al., 2020). However, it is difficult to directly compare the immunogenicity of chloroplast-derived RGNNV-VLPs with that of VLP vaccines expressed in *E. coli* or yeast. This is because the conditions for each virus challenge test are different, including the species and age of the fish, dose, frequency, interval of administration of the VLP vaccines, and the titer of the virus that infected the fish (Cho et al., 2017; Chien et al., 2018).

The RGNNV challenge of sevenband grouper vaccinated by intraperitoneal injection demonstrated that crudely purified protein extracts containing chloroplast-derived RGNNV-VLPs (RPS = 95.1) were more immunogenic than the commercial inactivated vaccine (RPS = 85.0) (Figure 7 and Table 1). This result is consistent with the induction of extremely high levels of neutralizing antibodies against RGNNV in the fish group vaccinated with chloroplast-derived RGNNV-VLPs (Supplementary Figure S2). Furthermore, oral administration of chloroplast-derived RGNNV-VLPs also showed significant immunogenicity (RPS = 72.4), although it was lower than that of parenteral administration. However, the variables influencing oral immunization (dose, number of doses, and administration interval) have not been optimized, and future studies are needed to improve the immunogenicity of orally administered chloroplast-derived RGNNV-VLPs. It should also be noted that the levels of neutralizing antibodies elicited by oral administration of chloroplast-derived RGNNV-VLPs and by injection of a commercially available inactivated vaccine were lower than expected (Supplementary Figure S2), and the results could not clearly explain the high RPS values in the RGNNV challenge test (Figure 7 and Table 1). Similar contradictory results were reported when sea bass was immunized by injection of ACNNV-VLPs transiently expressed in tobacco leaves. In a previous study, parenteral administration of plant-derived VLPs to fish provided some protection against NNV, but failed to induce statistically significant anti-NNV antibodies (Marsian et al., 2019). One hypothesis to explain these seemingly contradictory results is that injection or oral administration of an inactivated or VLP vaccine against NNV in fish may activate the humoral and cellular immune systems simultaneously. This is because it has been reported that immunization of fish with inactivated or VLP-based vaccines not only induces neutralizing antibodies against NNV, but also activates the expression of genes associated with cellular immunity against betanodavirus infection (Kai et al., 2014; Lai et al., 2014).

Immersion vaccination is an alternative immunization method other than injection or oral administration and is suitable for mass vaccination of fish that are too small for vaccination by injection (Bøgwald and Dalmo, 2019). Immersion vaccination of fish with OSGNNV-VLPs derived from *E. coli* has been reported to be more immunogenic than injection, however, the dose of OSGNNV-VLPs required to confer significant protective immunity by immersion was approximately 125 times higher than that of injection (Chien et al., 2018), making it difficult to reduce the cost of the vaccine. Although immersion vaccination of fish with chloroplast-derived RGNNV-VLPs has not been investigated, the high yield of RGNNV-VLPs produced by transplastomic tobacco plants in this study suggests that immersion administration of chloroplast-derived RGNNV-VLPs may be an affordable vaccination method.

In this study, we have shown that chloroplast genetic engineering is a promising platform for the mass production of highly immunogenic RGNNV-VLP vaccines. However, it will not be easy to commercialize the transplastomic plants developed in the present study. Crude protein extracts from tobacco leaves contain alkaloids, such as nicotine, which must be removed before vaccination, but the cost of the purification process is thought to be an obstacle to practical application. Even though the use of low-alkaloid tobacco as a host plant is possible, given consumer psychology, the use of a non-edible plant as an oral vaccine may not be socially acceptable. One possible solution to this problem is to develop transplastomic plants that express large amounts of RGNNV-CP using leafy greens as host plants. Lettuce is a representative leafy vegetable for which a reproducible chloroplast transformation method has been established. Lettuce transplastomic plants expressing various recombinant antigens derived from human and animal pathogens have been developed. Some studies have demonstrated that recombinant antigens can be maintained in lyophilized transgenic lettuce leaves for a long time at ambient temperature, and specific antibodies against the antigens were induced in mice by oral administration of lyophilized plant materials (Davoodi-Semiromi et al., 2010; Lakshmi et al., 2013; Daniell et al., 2019). Fortunately, it has been reported that RGNNV-VLPs can maintain their immunogenicity even after lyophilization (Lan et al., 2018). Therefore, lyophilized powder of transplastomic lettuce leaves expressing RGNNV-VLPs mixed with feed pellets and orally administered to fish would be an ideal method of VNN vaccination. We are currently working on the generation of transplastomic lettuce plants overexpressing RGNNV-CP.

In the present study, we successfully expressed RGNNV-VLPs with sufficient immunogenicity in tobacco chloroplasts, suggesting that plastid transformation may be effective for mass production of VLP vaccines against other fish diseases caused by non-enveloped viruses, such as infectious pancreatic necrosis virus (IPNV) (Jeong et al., 2020). The same is likely to be true for the manufacture of VLP vaccines for the prevention of viral diseases affecting humans and animals. While most reports on the production of VLP vaccines in plants have utilized stable nuclear transformation or transient expression systems (Marsian and Lomonossoff, 2016; Rybicki, 2020), the use of chloroplast genetic engineering may also be worth considering.

Since VLPs are particulate and have a high density of capsid proteins on their surface, attempts have been made to display heterologous antigens on the surface of VLPs by genetic fusion or chemical conjugation to generate more immunogenic vaccines (Chen and Lai, 2013; Rybicki, 2020). RGNNV-VLPs may serve as attractive carriers for displaying foreign antigens. This is because it has been reported that when foreign peptides were fused to the C-terminus of OGNNV-CP, which is 100% identical in amino acid sequence to RGNNV-CP, and expressed in *E. coli*, the modified OGNNV-CPs were able to self-assemble into stable VLPs displaying a high density of foreign peptides (Xie et al., 2016). If similar improvements can be applied to chloroplast-expressed RGNNV-VLPs, they could provide a versatile platform for the development of affordable vaccines against various fish diseases caused by enveloped viruses, bacteria, protozoa, and metazoans. Furthermore, a previous study by another group reported that oral administration of lyophilized powder of transplastomic tobacco leaves expressing NNV-CP induced significant NNV-CP-specific IgA and IgG responses in immunized mice (Cho et al., 2018), suggesting that chloroplast-derived RGNNV-VLPs may also play a promising role in disease prevention in mammals, including humans.

## Conclusion

The results of the present study show that chloroplast genetic engineering is a promising platform for the high-yield production of RGNNV-VLPs, which can confer high immunity to fish by injection or oral administration. In the future, if leafy vegetables such as lettuce overproducing RGNNV-VLPs can be generated by plastid transformation, they could be commercialized as ideal oral vaccines that are cost-effective, labor-saving, and fish-friendly. Further research on the production and improvement of VLPs using chloroplast genetic engineering, not limited to RGNNV-VLPs, may provide an impetus for the development of affordable oral vaccines to prevent infectious and chronic diseases in animals and humans.

## Data Availability Statement

The original contributions presented in the study are included in the article/Supplementary Material, and further inquiries can be directed to the corresponding author.

## Author Contributions

YN made substantial contributions to the conception and design of this work, the generation and analysis of transgenic plants, the interpretation of the results, and the preparation of the manuscript. TS and MT helped in the generation and analysis of transgenic plants, respectively. KT performed the expression and purification of *E. coli*-derived RGNNV-VLPs and TEM observations of the purified VLPs. KM, HY, and HK conducted fish experiments. All authors approved the final version of the manuscript.

## Conflict of Interest

The authors declare that the research was conducted in the absence of any commercial or financial relationships that could be construed as a potential conflict of interest.

## Publisher’s Note

All claims expressed in this article are solely those of the authors and do not necessarily represent those of their affiliated organizations, or those of the publisher, the editors and the reviewers. Any product that may be evaluated in this article, or claim that may be made by its manufacturer, is not guaranteed or endorsed by the publisher.

## Supplementary Material

Supplementary Figure S1 To examine the immunogenicity of chloroplast-derived RGNNV-VLPs, crudely purified protein extracts were prepared from wild-type (WT) or the transplastomic (RGNNV1-2) tobacco plants. TSP extracted from mature leaves of WT or RGNNV1-2 plants was ultrafiltrated using a centrifugal filter device to reduce the contamination of alkaloids, such as nicotine. The protein samples (10 μg) before ultrafiltration (Crude) and after ultrafiltration (Ultrafiltrated) were separated by 13% SDS-PAGE and stained with CBB.

Supplementary Figure S2 Anti-RGNNV neutralizing titers in sevenband grouper immunized with chloroplast-derived RGNNV-VLPs. Fish were vaccinated with PBS as control, crudely purified protein extracts from mature leaves of wild-type (WT) or the transplastomic (RGNNV1) tobacco plants, or a commercial inactivated vaccine (Inactivated) by intraperitoneal injection (Injection) or oral administration (Oral). After 21 days post-immunization, sera were collected from the caudal veins of fish (*n* = 8 for PBS-Injection and WT-Oral; *n* = 9 for Inactivated-Injection; *n* = 10 for WT-Injection, RGNNV1-Injection, PBS-Oral, and RGNNV1-Oral). Titer 0 indicates 1:80 or lower. ^*^*p* < 0.01; ^**^*p* < 0.05.
