# Supplementary figures and images for: Mass Production of Virus-Like Particles Using Chloroplast Genetic Engineering for Highly Immunogenic Oral Vaccine Against Fish Disease
Source: Front Plant Sci. 2021 Aug 23;12:717952. doi: 10.3389/fpls.2021.717952 (PMC8419230; doi:10.3389/fpls.2021.717952)

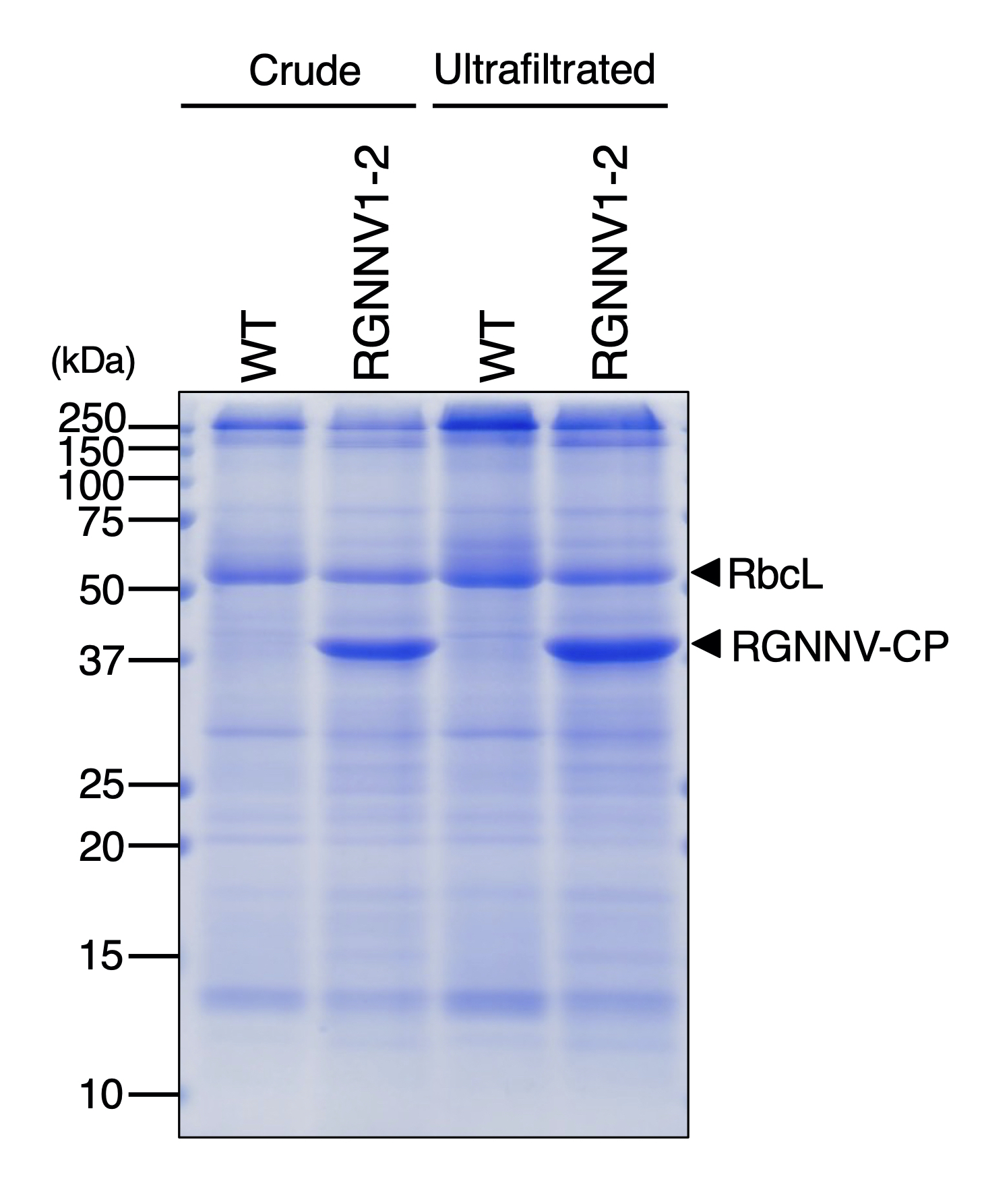

Supplement: Supplementary Figure S1 — To examine the immunogenicity of chloroplast-derived RGNNV-VLPs, crudely purified protein extracts were prepared from wild-type (WT) or the transplastomic (RGNNV1-2) tobacco plants. TSP extracted from mature leaves of WT or RGNNV1-2 plants was ultrafiltrated using a centrifugal filter device to reduce the contamination of alkaloids, such as nicotine. The protein samples (10 μg) before ultrafiltration (Crude) and after ultrafiltration (Ultrafiltrated) were separated by 13% SDS-PAGE and stained with CBB. [file Image_1.JPEG]

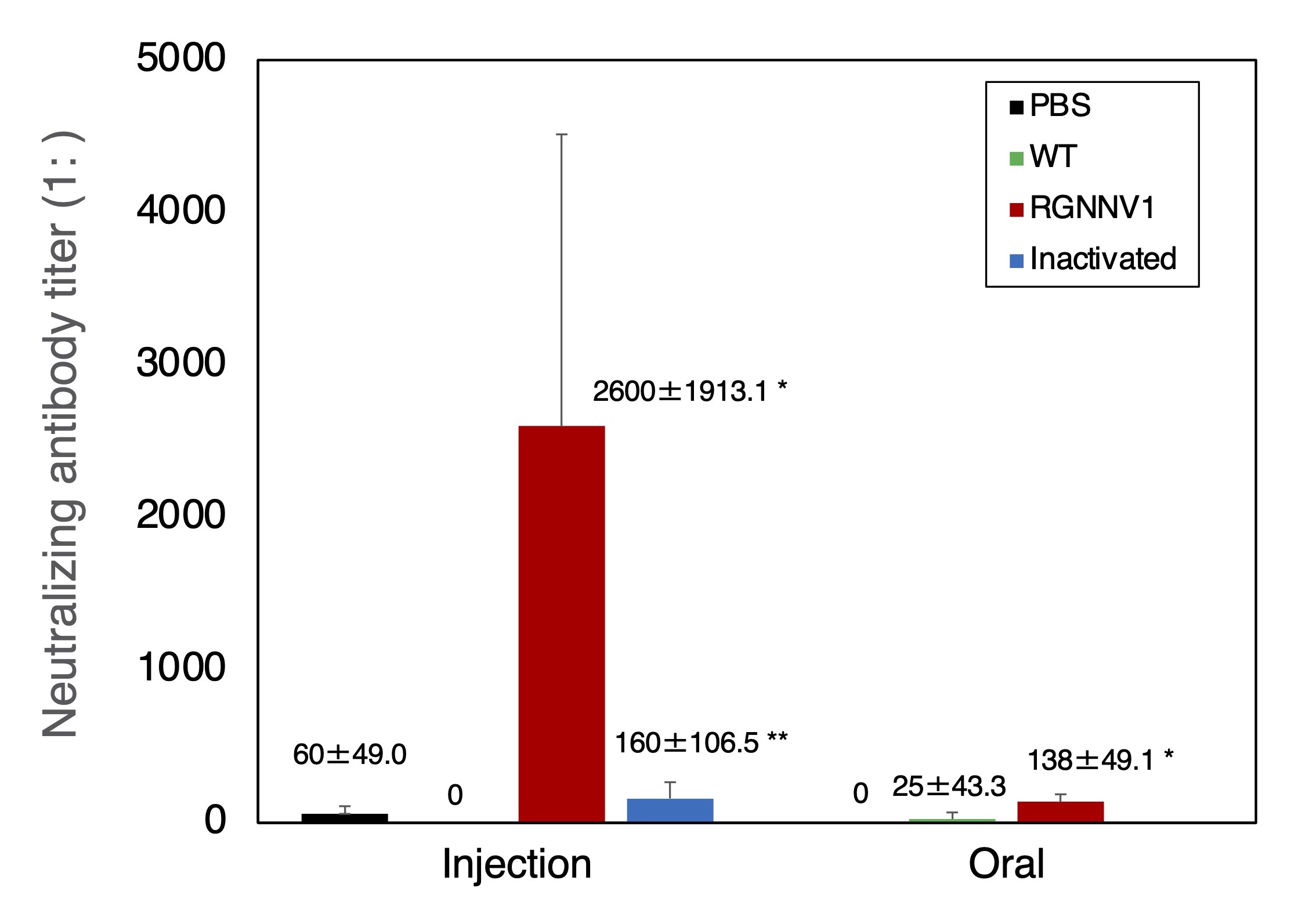

Supplement: Supplementary Figure S2 — Anti-RGNNV neutralizing titers in sevenband grouper immunized with chloroplast-derived RGNNV-VLPs. Fish were vaccinated with PBS as control, crudely purified protein extracts from mature leaves of wild-type (WT) or the transplastomic (RGNNV1) tobacco plants, or a commercial inactivated vaccine (Inactivated) by intraperitoneal injection (Injection) or oral administration (Oral). After 21 days post-immunization, sera were collected from the caudal veins of fish (n = 8 for PBS-Injection and WT-Oral; n = 9 for Inactivated-Injection; n = 10 for WT-Injection, RGNNV1-Injection, PBS-Oral, and RGNNV1-Oral). Titer 0 indicates 1:80 or lower. *p < 0.01; **p < 0.05. [file Image_2.JPEG]
